# Supplementary figures and images for: Advances in geometric techniques for analyzing blebbing in chemotaxing Dictyostelium cells
Source: PLoS One. 2019 Feb 14;14(2):e0211975. doi: 10.1371/journal.pone.0211975 (PMC6375592; doi:10.1371/journal.pone.0211975)

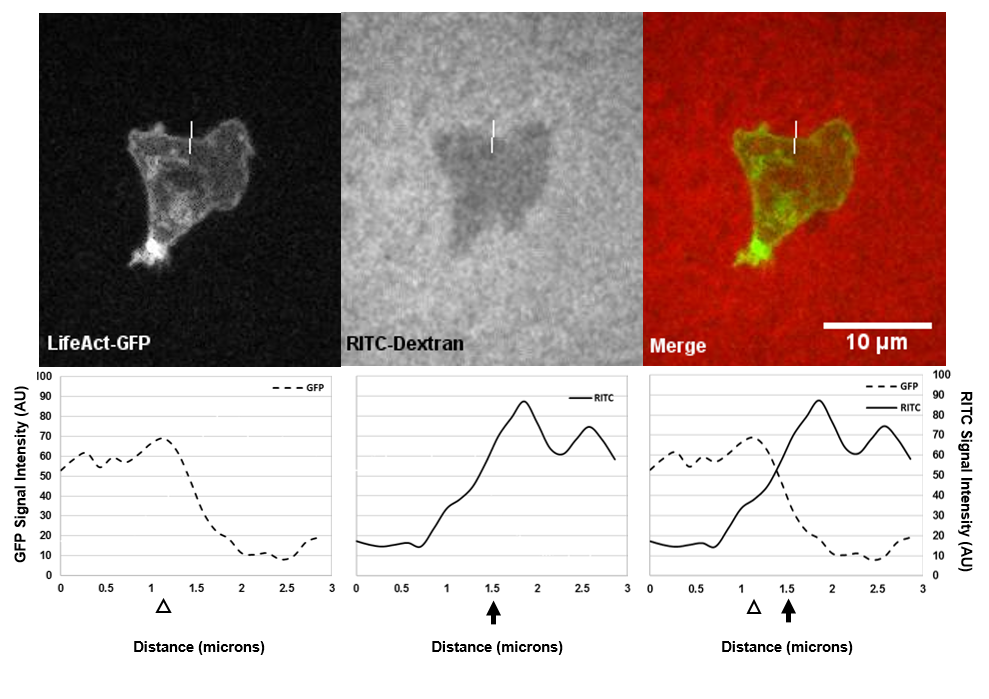

Supplement: S1 Fig — Using the same frame of the cell in Fig 2, intensity plots of the signals along the drawn line (white) for each channel show that in a region without a bleb, the membrane and cortex are observed to be in close proximity. Cortex and membrane positions are indicated by (white arrow head) and (black arrow), respectively. (TIF) [file pone.0211975.s001.tif]

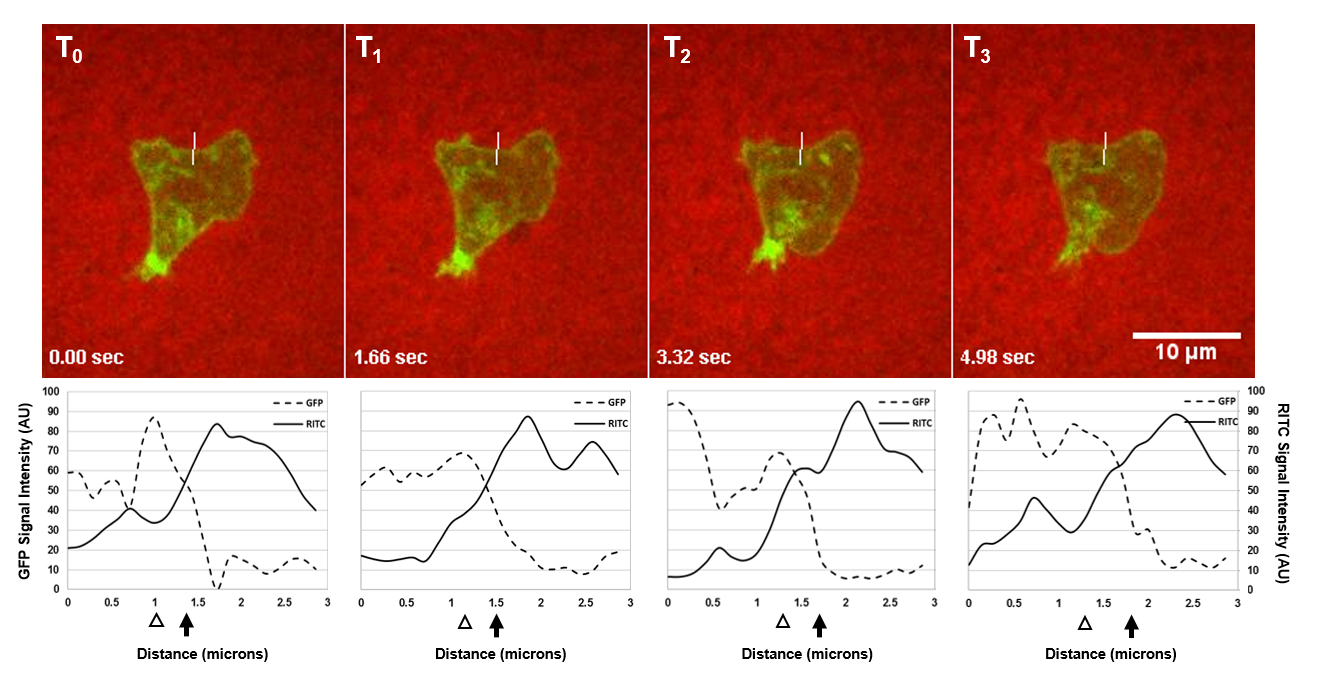

Supplement: S2 Fig — Using the same frames of the cell in Fig 4, intensity plots were made of a region where there was no bleb (white line). Merges of GFP and RITC time points (T0 − T3 for bleb characteristics) with their respective intensity plots (dashed and solid lines) confirmed a sustained cortex-to-membrane complex over time. Cortex and membrane positions are indicated by (white arrow head) and (black arrow), respectively. (TIF) [file pone.0211975.s002.tif]

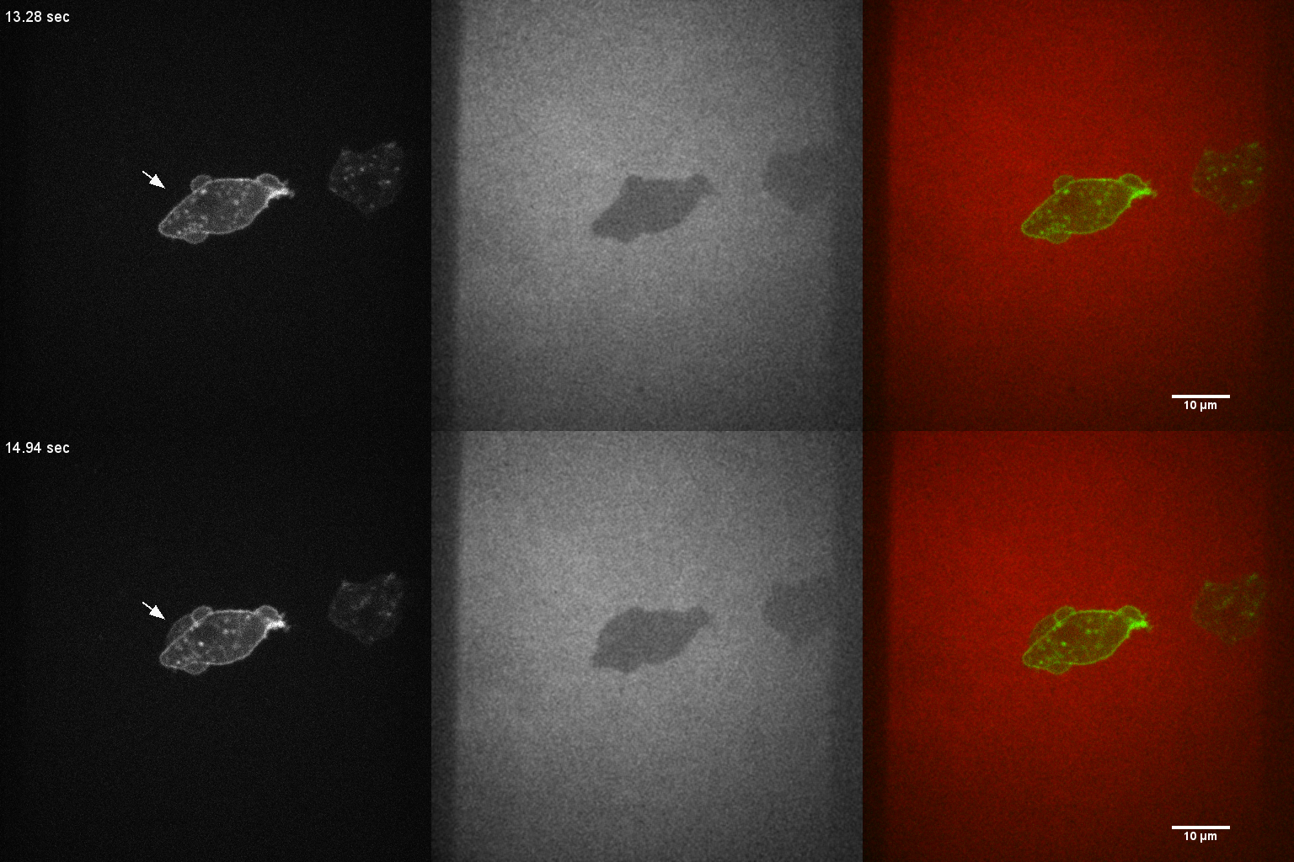

Supplement: S3 Fig — Top row shows the frame before the bleb, and the bottom row shows the frame with the bleb in progress, identified by the actin debris present in the bleb. Left, in the GFP channel, the white arrow indicates the region where the actin debris will be present in both time frames. Center, the RITC channel shows membrane position in both frames with the bleb region present in the second time frame. Right, the merge between both GFP and RITC channels false colored green and red, respectively. Images were taken on the 100x objective. (TIF) [file pone.0211975.s003.tif]

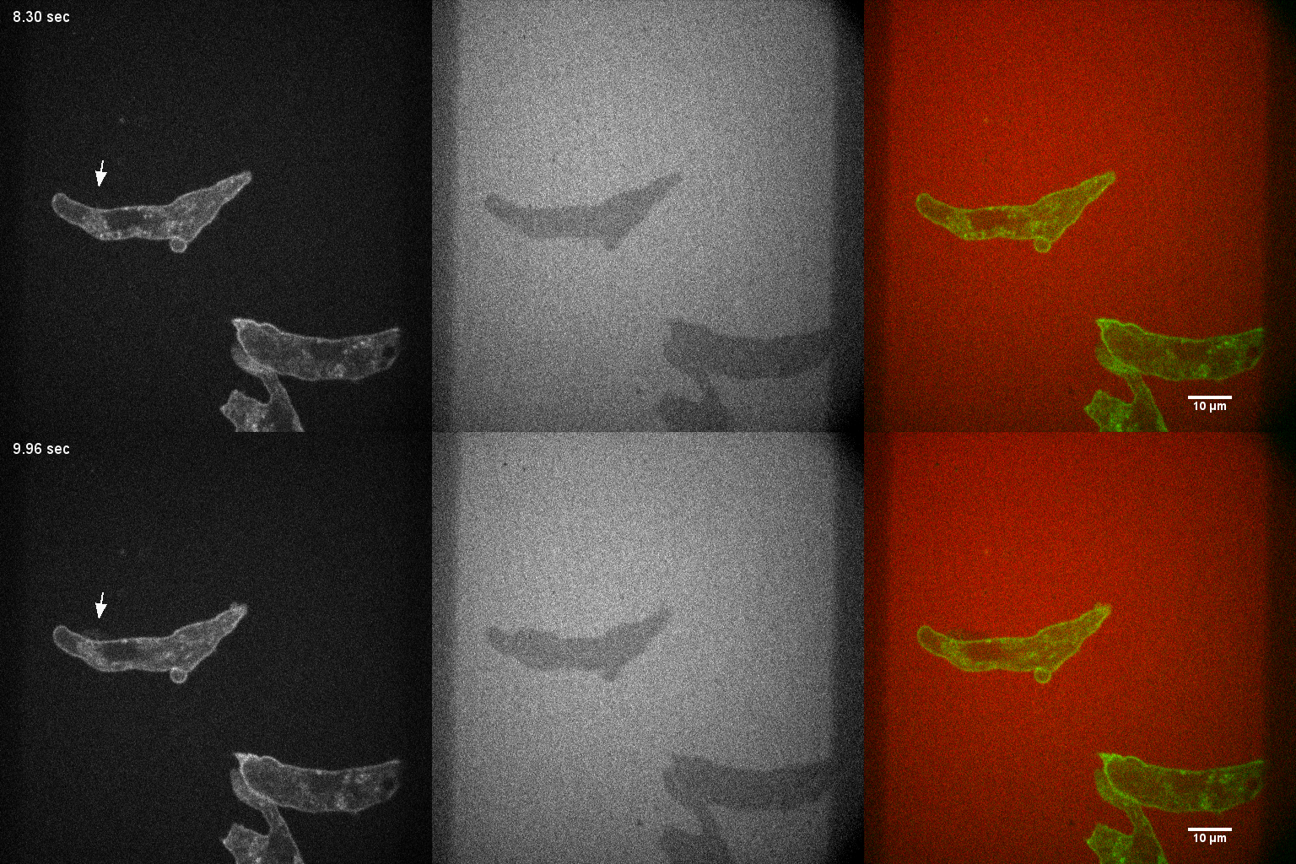

Supplement: S4 Fig — Top row shows the frame before the bleb, and the bottom row shows the frame with the bleb in progress, identified by the actin debris present in the bleb. Left, in the GFP channel, the white arrow indicates the region where the actin debris will be present in both time frames. Center, the RITC channel shows membrane position in both frames with the bleb region present in the second time frame. Right, the merge between both GFP and RITC channels false colored green and red, respectively. Images were taken on the 80x objective. (TIF) [file pone.0211975.s004.tif]

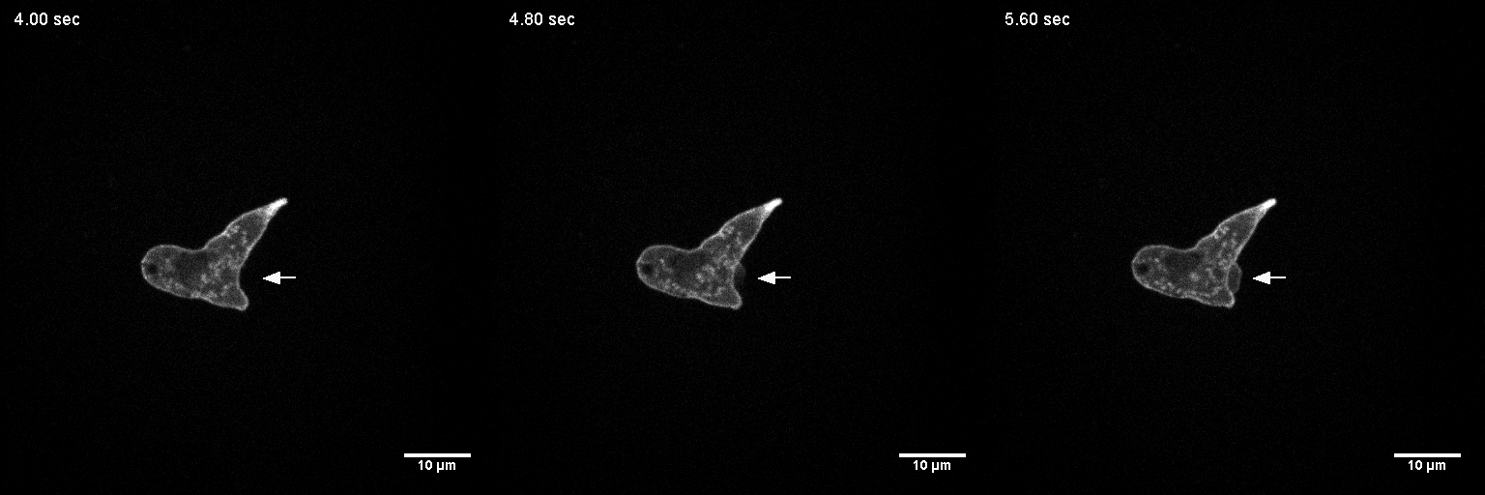

Supplement: S5 Fig — Left, the frame before the bleb. Center, the frame with the bleb in progress, identified by the actin debris present in the bleb. Right, the next frame showing the cortex reforming in the bleb. Images were taken on the 100x objective. (TIF) [file pone.0211975.s005.tif]
