# Supplementary material for: Advances in geometric techniques for analyzing blebbing in chemotaxing Dictyostelium cells
Source: PLoS One. 2019 Feb 14;14(2):e0211975. doi: 10.1371/journal.pone.0211975 (PMC6375592; doi:10.1371/journal.pone.0211975)
Supplement: S1 Appendix — More technically, we prove that as we add more guide points on the cortex, the resulting B-spline better approximates the cortex curve. (PDF) [file pone.0211975.s010.pdf]

S1 Appendix: Convergence Result for B-Splines

See [25] for a development of B-Splines. The following argument is outlined in that reference.

Let f be a function defined on an interval $[a, b]$. f is Lipschitz continuous provided there is a constant K with $|f(c) - f(d)| < K|c - d|$ for any $c, d \in [a, b]$. We want to prove the following statement:

Let f be Lipschitz continuous, real valued function on an interval, $[a, b]$ and let $a = \alpha_0 < \alpha_1 < \ldots < \alpha_n = b$ be a partition of the interval with mesh parameter, $h = \max_i(\alpha_i - \alpha_{i-1})$. Suppose that σ is a B-spline supported by the point set $(\alpha_i, f(\alpha_i))$, then σ converges to f in L^∞ norm as $h \to 0$. In particular, if $(x, f(x))$ is a point on the graph of f and (x, y) is the corresponding point on σ, then $\|(x, f(x)) - (x, y)\| < 12Kh$, where K is the Lipschitz constant.

Let $\beta_i(t)$ be a B-spline basis function $(i = 1, 2, 3, 4)$ and set $L = \max_{i,t} |\beta_i(t)|$, for t in $[0, 1]$. It is routine to verify that $L < 1$.

We begin convergence. We take x in $[a, b]$ and suppose that x is the first coordinate of a point on the B-spline. In particular, there is a k so that x associates to the kth B-spline segment, σ_k. Hence, there is a t_0 in the unit interval with the first coordinate of $\sigma_k(t_0)$ equal to x. Let y denote the second coordinate. In addition, if γ is the first coordinate of $\sigma_k(0)$ and δ is the corresponding coordinate of $\sigma_k(1)$, then

$$\alpha_k < \frac{1}{6}\alpha_k + \frac{2}{3}\alpha_{k+1} + \frac{1}{6}\alpha_{k+2} = \gamma < x < \delta = \frac{1}{6}\alpha_{k+1} + \frac{2}{3}\alpha_{k+2} + \frac{1}{6}\alpha_{k+3} < \alpha_{k+3}.$$

Therefore, $|x - \alpha_{k+j}| \leq 3h$ for $j = 0, 1, 2, 3$.

We verify $\|(x, f(x)) - (x, y)\| < 12KLh$ in Euclidean norm. Toward this end, we calculate,

$$\|(x, f(x)) - (x, y)\| = |f(x) - y| = |f(x) - \sum_{j=0}^{3} \beta_j(t_0) f(\alpha_{k+j})| =$$

$$|\sum_{j=0}^{3} \beta_j(t_0) f(x) - \sum_{j=0}^{3} \beta_j(t_0) f(\alpha_{k+j})|,$$

since $\sum_{j=0}^{3} \beta_j(t) = 1$. Continuing,

$$\|(x, f(x)) - (x, y)\| = |\sum_{j=0}^{3} \beta_j(t_0)[f(x) - f(\alpha_{k+j})]| \leq$$

$$\sum_{j=0}^{3} |\beta_j(t_0)| K |x - \alpha_{k+j}| \leq LK \sum_{j=0}^{3} |x - \alpha_{k+j}| < 12Kh,$$

since $x \in [\alpha_k, \alpha_{k+3}]$. The proposition now follows.
